# Abundant immunoglobulin E-positive cells in skin lesions support an allergic etiology of atopic dermatitis in the elderly

**DOI:** 10.1111/j.1468-3083.2012.04612.x

**Published:** 2013-08

**Authors:** R Tanei, Y Hasegawa, M Sawabe

**Affiliations:** † Departments of Dermatology, Tokyo Metropolitan Geriatric Hospital and Institute of GerontologyTokyo, Japan; ‡ Bioresource Center for Geriatric Research, Tokyo Metropolitan Geriatric Hospital and Institute of GerontologyTokyo, Japan

## Abstract

**Background/Objectives:**

Atopic dermatitis (AD) in the elderly is gradually increasing in industrialized countries in association with the aging of society. We report herein four cases of elderly AD {three extrinsic [immunoglobulin (Ig)E-mediated allergy]; one intrinsic (non-IgE-allergy)} in which we investigated the presence of IgE+ cells in lesional skin.

**Methods/Results:**

Single immunohistochemical and double immunofluorescence stainings were performed for skin biopsy specimens from AD patients and non-atopic control subjects with chronic eczema. In the lesional lichenified skin of patients with extrinsic elderly AD, numerous IgE+ cells were found among inflammatory cells infiltrates in the upper dermis. Comparative analysis of single immunohistochemistry results using serial paraffin and/or frozen sections found that many IgE+ cells showed identical distributions to tryptase+ mast cells. IgE+ cells coincident with CD1a+ Langerhans cells in the epidermis were found in small numbers only in frozen sections. Double immunofluorescence staining for IgE and CD11c revealed cells coexpressing IgE and CD11c with a dendritic morphology in the papillary and upper dermis. These IgE+ mast cells and IgE+ CD11c+ cells were also found in cured normal-looking skin from a patient with extrinsic elderly AD after successful treatment. Although only a few weakly positive IgE+ cells were detected, no IgE+CD11c+ cells were found in specimens from patients with intrinsic elderly AD or non-atopic chronic eczema.

**Conclusion:**

IgE-mediated allergic inflammation may play an important role in the pathobiology of elderly AD, similar to other age groups of AD.

## Introduction

Atopic dermatitis (AD) has been ordinarily divided into infantile, childhood and adolescent/adult types according to the age of the patient and the characteristics of typical skin lesions. However, the number of elderly patients with AD has been gradually increasing in industrialized countries in parallel with the aging of society, and a new subgroup of elderly AD has been reported and characterized in recent reports.^1^–^4^ Three main patterns of onset are present in elderly AD: geriatric onset; geriatric recurrence of classic childhood AD; and geriatric recurrence and/or continuation of adult AD. Similar to AD in other age groups, both immunoglobulin (Ig)E-mediated allergic (extrinsic) and non-IgE-allergic (intrinsic) forms exist in elderly AD, and the most frequent environmental allergens involved in the extrinsic form are house dust mites (e.g. *Dermatophagoides* species), followed by pollens and foods.^1^,^4^ Skin manifestations in elderly AD basically match those of adult AD, although the reverse sign of lichenified eczema around unaffected folds of the elbows and knees is more common than the classic sign of localized lichenification in those folds.^1^,^2^

Although the clinical features of elderly AD have been largely characterized, some issues remain to be addressed in this subgroup. First, the diagnosis of elderly AD is difficult, since elderly individuals often have pruritic skin disorders, e.g. asteatotic dermatitis, senile pruritus, chronic prurigo and adverse drug reactions, which have similar skin manifestations to elderly AD.^2^ More specific and objective methods are thus needed for the diagnosis of elderly AD in addition to the standard clinical criteria. Second, the pathological mechanisms underlying elderly AD should be analysed to identify whether IgE-mediated allergy plays an important role or is just an incidental finding in the immunopathogenesis of elderly AD.

To address these issues, we performed immunohistochemical and double immunofluorescence studies for infiltrating IgE+ cells in skin lesions from four typical cases of elderly AD, and thus analysed the allergic etiology of elderly AD.

## Subjects and methods

### Diagnosis of AD and laboratory examinations

AD was diagnosed according to the clinical criteria of Hanifin and Rajka^5^ for four Japanese patients, and the severity of AD was scored using the Severity Scoring of Atopic Dermatitis (SCORAD) index.^6^ Serum levels of specific IgEs against environmental allergens were detected using the multiple antigen simultaneous test (MAST)-26^1^ and the revised MAST-33 version (BML, Tokyo, Japan). Allergic skin tests (e.g. prick tests and atopy patch tests) were not performed, since most patients had been receiving standard anti-inflammatory treatments (e.g. topical corticosteroids and oral antihistamines)^7^ prescribed by their family doctors. Such treatments would have suppressed skin reactivity tests at the first medical examination in our hospital, and patients did not wish to discontinue these medical treatments for skin tests.

### Skin samples

Skin biopsy specimens were obtained from lichenified skin lesions of Cases 1–4 and from cured normal-appearing skin of Case 3 after 10 years treatment. As controls, four non-atopic skin samples were obtained from two elderly patients with chronic eczema (asteatotic dermatitis and nummular dermatitis) and two volunteers with normal skin. As another control, a skin sample was also obtained from a patient with cholesterol embolism and serum hyper-IgE (Table 1: AD, Case 1–4; controls, Cases 5–9). Serial 3-μm-thick sections from formalin-fixed, paraffin-embedded specimens were used for immunohistochemical staining. Frozen sections, except for those of a lichenified lesion in Case 3 and control Cases 8 and 9, were used for the single immunohistochemical and double immunofluorescence stainings for IgE, CD11c and CD1a. All study protocols were approved by the Ethics Committee of the Tokyo Metropolitan Geriatric Hospital and Institute of Gerontology. AD patients and control subjects all provided written informed consent for every biopsy and research use of specimens.

**Table 1:** Clinical and laboratory data for elderly patients with atopic dermatitis and control subjects

Case	Age/sex	Skin condition	Biopsy site	Allergic form	Onset type*	Topical steroid treatment†	WBC (/mm^3^)‡/eosinophils (%)‡	Total IgE (IU/mL)‡	Allergen-specific IgEs (major allergens)§	SCORAD
1	79/M	AD	Upper back	Extrinsic	I	Not use	7360/22.2	10429	Der. f, shrimp, crab	40.6
2	71/M	AD	Upper arm	Extrinsic	II	Medium, 10 yr.	7130/14.0	2413	Der. f, Japanese cedar	64.2
3	73/M	AD	Thigh	Extrinsic	III	Strong, 7 mo.	15 380/30.0	15238	Der. f	86.1
3¶	83/M	AD	Back	(Extrinsic)	(III)	Very strong, 10 yr.	NT	208	Normal levels of all allergens	8.4
4	81/F	AD	Forearm	Intrinsic	I	Very strong, 7 yr.	8800/2.0	5	Normal levels of all allergens	88.8
5	86/M	AS	Back			Very strong, 2 mo.	6610/NT	16	Normal levels of all allergens	36.7
6	87/F	ND	Back			Strong, 5 mo.	3640/8.0	47	Normal levels of all allergens	37.8
7	83/F	NS	Buttocks			Not used	3740/2.9	18	Normal levels of all allergens	0.0
8	75/M	NS	Back			Not used	9060/1.3	123	Normal levels of all allergens	0.0
9	75/M	CE	Foot			Not used	10 960/57.8	38155	Four minor allergens only	0.0

* Onset type: I, geriatric onset; II, geriatric recurrence of classic childhood AD; III, geriatric recurrence and/or continuation of adult AD.

† Main rank of using topical corticosteroids in the treatment in accordance with a guideline^7^ and the periods before skin biopsy.

‡ Normal range: WBC, 4800–7500 mm^3^; eosinophils, 2.0–4.0%; IgE, ≤400 IU/mL.

§ Allergens in class 3 for multiple antigen simultaneous test (MAST)-26 or in class 4, 5 or 6 for MAST-33.

¶ Taken at the cured point after 10-year treatment.

AD, atopic dermatitis; AS, asteatotic dermatitis; CE, cholesterol embolism; Der. f, *Dermatophagoides farinae*; Extrinsic, extrinsic (IgE-mediated allergic) form; F, female; Intrinsic, intrinsic (non-IgE-allergic) form; M, male; mo., months; ND, nummular dermatitis; NS, normal skin; NT, not tested; SCORAD, Severity Scoring of Atopic Dermatitis (maximum, 103)^6^; WBC, white blood cell count; yr., years.

### Histological and immunohistochemical staining

Haematoxylin and eosin (HE) and Giemsa stainings were performed to evaluate routine histopathological findings. Immunohistochemical single stainings were performed using the streptavidin-biotin method with an LSAB kit (Dako, Tokyo, Japan), as previously reported.^8^ The following primary monoclonal antibodies (mAb) were applied: mouse mAb against CD4 (helper/inducer T cells, #713181; Nichirei, Tokyo, Japan), CD8 (cytotoxic/suppressor T cells, #713201; Nichirei), CD20 (B cells, L26; Nichirei), CD68 (macrophages, N1576; Dako), CD1a (Langerhans cells/dendritic cells, NCL-CD1a-235; Novocastra, Newcastle, UK), mast cell tryptase (mast cells, AA1; Abcam, Tokyo, Japan), and IgE ε-chain (IgE; MH25-1; Santa Cruz Biotechnology, Santa Cruz, CA, USA); and rabbit mAb to CD11c (dermal dendritic cells, EP1347Y; LSBio, Seattle, WA, USA). The degree of staining was semiquantitatively graded according to staining intensity and numbers of positively staining cells as: ‘negative’, −; ‘weakly positive’, ±; ‘positive’, +; or ‘strongly positive’, ++. A dermatologist (R.T.) blinded to specimen identification analysed all sections.

### Double immunofluorescence staining

Double immunofluorescence studies were performed using frozen sections fixed in cold acetone. Combinations of primary and secondary antibodies were as follows: biotinylated anti-mouse IgG (BA-9200; Vector, Burlingame, CA, USA) – anti-IgE mouse mAb (MH25-1; Santa Cruz); and biotinylated anti-rabbit IgG (BA-1000; Vector) – anti-CD11c rabbit mAb (EP1347Y; LSBio). The fluorescein-conjugated streptavidins used were DyLight488 streptavidin (SA-5488; Vector) and DyLight594 streptavidin (SA-5594; Vector). Dilution ratios for primary antibodies, secondary antibodies, and fluorescein-conjugated streptavidin were as follows: ×200 for anti-IgE; ×250 for anti-CD11c; ×150 for biotinylated anti-IgG; and ×100 for streptavidin. Nuclei were stained with 4’,6-diamidino-2-phenylindole (DAPI). Sections were viewed under fluorescence microscopy for ultra-fast 2-dimensional (2D) time-lapse experiments and ratio imaging (Leica AF6500; Leica Microsystems, Wetzlar, Germany).

## Results

Clinical and laboratory data for AD patients and controls are summarized in Table 1. Results of immunohistochemical and double immunofluorescence studies are summarized in Table 2.

**Table 2:** Immunohistochemical and double immunofluorescence staining results for analyses of IgE-bearing cells in atopic dermatitis and control subjects

Case	Disease	Immunohistochemistry						Double immunofluorescence					
		(paraffin section)				(frozen section)		(frozen section)					
		Tryptase^+^ mast cells	IgE^+^ cells		CD1a^+^ dendritic cells	IgE^+^ cells		IgE^+^ cells*		IgE^+^CD11c^+^ cells†		CD11c^+^ cells*	
		Dermis	Dermis	Epidermis	Epidermis	Dermis	Epidermis	Dermis	Epidermis	Dermis	Epidermis	Dermis	Epidermis
1	AD	+	++	–	+	++	+	++	+	++	–	++	+
2	AD	++	++	–	+	++	+	++	±	++	±	++	±
3	AD	++	++	–	+	NA	NA	NA	NA	NA	NA	NA	NA
3‡	AD	+	+	–	+	+	–	++	±	+	–	+	±
4	AD	+	±	–	±	±	–	±	–	–	–	++	±
5	AS	+	±	–	+	±	–	±	–	–	–	+	±
6	ND	+	±	–	++	±	–	+	–	–	–	++	±
7	NS	+	±	–	+	±	–	–	–	–	–	±	–
8	NS	±	±	–	+	NA	NA	NA	NA	NA	NA	NA	NA
9	CE	+	+	–	±	NA	NA	NA	NA	NA	NA	NA	NA

* Before merge.

† After merge.

‡ Taken at the cured point after 10-year treatment.

Semi-quantitative grade of staining: −, negative; ±, weakly positive; +, positive; ++, strongly positive.

AD, atopic dermatitis; AS, asteatotic dermatitis; CE, cholesterol embolism;

NA, not available; ND, nummular dermatitis; NS, normal skin.

### Case presentations

#### Case 1

**Figure 1:**
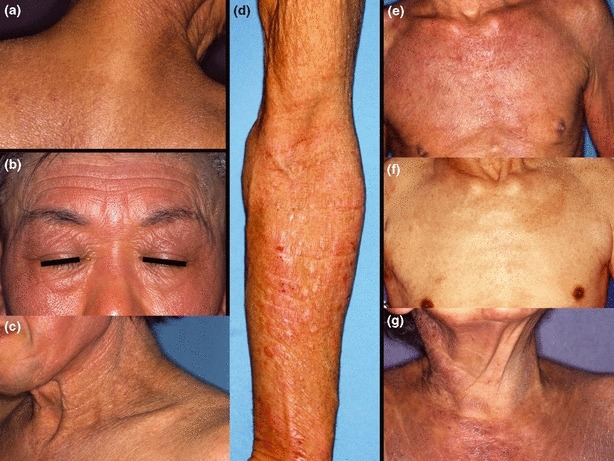
Skin manifestations of elderly AD: extrinsic form, Cases 1–3; intrinsic form, Case 4 (a) Lichenified eczema on the upper back (Case 1). (b) Facial erythema (atopic red face) with Hertogh’s sign (loss of lateral eyebrows) and Dennie-Morgan infraorbital folds (Case 2). (c) Dirty neck (Case 2). (d) Lichenified eczema around the scarcely involved elbow fold (reverse sign) (Case 2). (e) Eczematous erythroderma (Case 3). (f) Eczematous lesions mostly disappeared after successful treatment and the cured skin shows a normal appearance (Case 3). (g) Lichenified eczema of erythroderma on the trunk and neck (Case 4).

A 79-year-old man with hypertension, chronic kidney disease and senile depression complained of chronic eczema for 3 years. He showed mild eczematous dermatitis on the face and upper extremities and significant lichenified eczema on the nape and upper back (Fig. 1a). He also showed symptomatic allergic conjunctivitis, but had no family history of atopic disorders.

#### Case 2

A 71-year-old man who had a 10-year history of chronic eczema associated with allergic rhino-conjunctivitis was referred to our department with generalized erythroderma. He had a history of chronic eczema until 5 years old that matched the classic course of childhood AD, but no family history of atopic disorders was apparent. He showed facial erythema with Hertogh’s sign and Dennie-Morgan infraorbital folds (Fig. 1b), dirty neck (Fig. 1c) and diffuse lichenified eczema on the trunk and extremities. Lichenified eczema at the flexural sites of the upper extremities was mainly observed around the unaffected elbow folds (reverse sign)^1^,^2^ (Fig. 1d).

#### Case 3

A 73-year-old man with chronic eczema lasting 7 months was admitted to our department for erythrodemic eczematous rash. He had occasionally experienced chronic eczema since 29 years old, but had no personal or family history of atopic disorders. He showed facial erythema with Hertogh’s sign, and diffuse lichenified eczema on the trunk and extremities (Fig. 1e). He was treated with topical corticosteroids and moisturizer, and antihistamines, a T-helper (Th)2 cytokine inhibitor (suplatast tosilate,150 mg/day) and systemic corticosteroids (prednisolone, 15 mg/day) were administered. After 2 years of treatment, oral corticosteroids and Th2 cytokine inhibitor were discontinued after tapering of therapeutic doses. After 10 years of therapeutic management, most of the lesions had disappeared, replaced by normal-appearing skin (Fig. 1f). At this time, serum total IgE levels had decreased to within normal ranges and specific IgE was undetectable.

#### Case 4

An 81-year-old woman with a 7-year history of chronic eczema was referred to our departments with severe generalized erythroderma (Fig. 1g). She also showed facial pallor and the reverse sign of lichenification around the folds of the elbows and knees. She had experienced intrinsic asthma since she was 68 years old and reported family histories of asthma in her mother and aspirin-related urticaria in her son.

### Histological and immunohistochemical staining

#### Lichenified skin lesions

**Figure 2:**
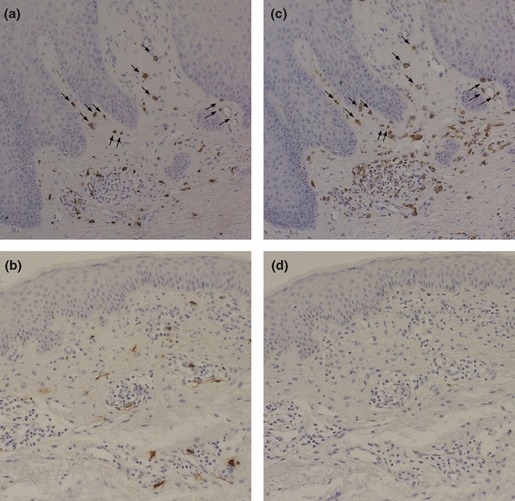
Single immunohistochemical staining with anti-mast-cell-tryptase antibody and with anti-IgE antibody using serial paraffin sections Sets of figures (a and c), and figures (b and d) represent serial sections. (a) Tryptase+ mast cell infiltration is increased in the papillary and upper dermis of a lichenified lesion from a patient with extrinsic elderly AD (Case 2). (b) Tryptase+ mast cells are scattered in a skin lesion from a patient with non-atopic chronic eczema (asteatotic dermatitis) (Case 5). (c) Numerous IgE+ cells are seen in inflammatory infiltrates of a lichenified lesion from a patient with extrinsic elderly AD (Case 2). (d) Few IgE+ cells are present in a skin lesion from a patient with non-atopic chronic eczema (Case 5). Note that, in specimens from cases with extrinsic elderly AD (a and c), most IgE+ cells and tryptase+ cells show the same morphology and localization (arrows). Original magnification: ×100.

Lichenified lesions of AD cases showed chronic eczematous reactions with infiltration of inflammatory cells, mainly comprising CD4 + , CD8 + , CD68 +  cells and eosinophils. A moderate number of CD1a+ dendritic cells, presumably Langerhans cells, had infiltrated the epidermis, and a few CD1a+ cells were present in the upper dermis. Tryptase+ mast cells had markedly infiltrated the papillary and upper dermis in Cases 1–3 (Fig. 2a). Giemsa staining showed that many of the mast cells in the papillary dermis presented as activated forms with an elongated shape and/or degranulated appearance. In control specimens of non-atopic chronic eczema (asteatotic dermatitis and nummular dermatitis), the composition and nature of infiltrating cells were basically the same as those of lichenified AD lesions, except without the increased mast cell infiltration (Fig. 2b).

Single immunolabeling using paraffin sections revealed numerous IgE+ cells among the inflammatory infiltrates in the upper dermis, but not in the epidermis, of lichenified lesions of AD in Cases 1–3. Most IgE+ cells were round, spindle-shaped or dendritic cells with IgE+ staining of the cell surface (Fig. 2c). Lesions in Case 4 and non-atopic chronic eczema showed only a few cells with weak positive staining for IgE (Fig. 2d). Comparative analysis of serial paraffin sections revealed that many of the IgE+ cells displayed the same morphology and localization as the infiltrating tryptase+ mast cells in Cases 1–3 (Fig. 2a and 2c). However, few of the IgE+ cells showed distributions topologically overlying those of CD4 + , CD8 + , CD68 + , and CD1a+ cells and eosinophils in the dermal infiltrates. In control Case 9 (cholesterol embolism with serum hyper-IgE), the vast majority of IgE+ cells were observed sporadically in accordance with the regular distribution of tryptase+ mast cells.

Using serial frozen sections, IgE+ cells present mainly in the papillary and upper dermis and partly in the epidermis showed distributions similar to those of CD11c+ cells in Cases 1 and 2. Small numbers of IgE+ cells in the epidermis were also observed with the same distribution as CD1a+ Langerhans cells in those cases.

#### Normal-appearing skin after treatments for AD

A biopsy specimen of cured skin from Case 3 after treatment for 10 years demonstrated normal histology with decreased inflammatory infiltrates. However, immunostaining of paraffin and frozen sections showed IgE+ cells, corresponding to tryptase+ mast cells or CD11c+ cells, still regularly present in the dermis.

### Double immunofluorescence staining

**Figure 3:**
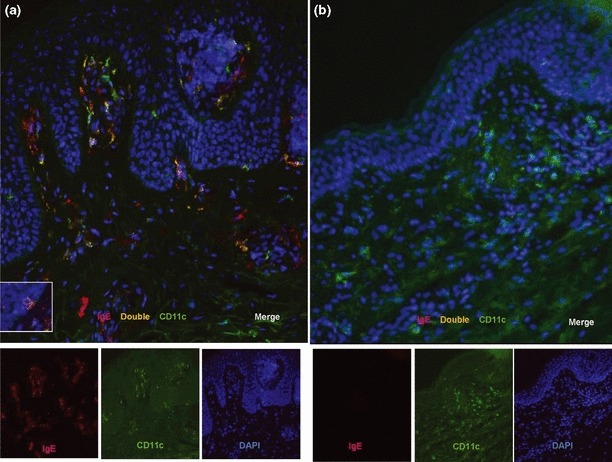
Double immunofluorescence staining with anti-IgE and anti-CD11c mAbs (a) This figure clearly shows three types of cells coexisting in the dermal papillae and upper dermis of lichenified skin lesions of a patient with extrinsic elderly AD (Case 2): single IgE+ cells (red images), mostly representing IgE-bearing mast cells; single CD11c+ cells (green images), representing dermal dendritic cells; and double-positive IgE+CD11c+ cells (yellow images), representing IgE-bearing dermal dendritic cells. The box in the upper panel indicates a dendritic IgE+CD11c+ cell in the basal layer of the epidermis. (b) Only single CD11c+ cells (green images) are apparent in a skin lesion from a patient with non-atopic chronic eczema (asteatotic dermatitis) (Case 5). Nuclei are labeled with DAPI (blue images). Original magnification: ×200.

**Figure 4:**
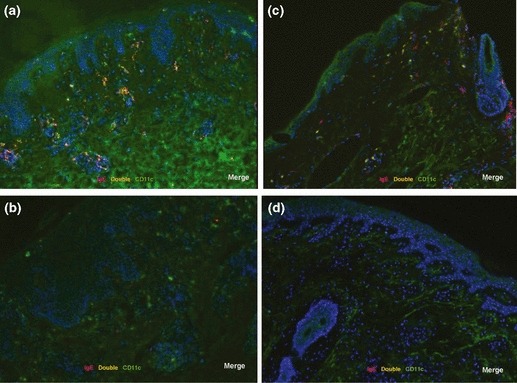
Double immunofluorescence staining with anti-IgE and anti-CD11c mAbs (a) Lichenified skin lesion from a patient with extrinsic elderly AD (Case 1). The majority of double-positive IgE+CD11c+ cells (yellow images) with a dendritic morphology are distributed in the papillary and upper dermis. Single IgE+ cells (red images) are seen in the upper dermis, but single CD11c+ cells (green images) are apparent in both the epidermis and dermis. (b) A lichenified skin lesion from a patient with intrinsic elderly AD (Case 4). Only a few single IgE+ cells (red images) and no double-positive IgE+CD11c+ cells are observed, although infiltrating single CD11c+ cells (green images) are seen in the upper dermis. (c) Cured normal-appearing skin of a patient with extrinsic elderly AD after successful treatment (Case 3). Cells coexpressing IgE and CD11c (yellow images) accompanied with single IgE+ cells (red images) and single CD11c+ cells (green images) are scattered among the dermis-infiltrating cells. Note that cells coexpressing IgE and CD11c (yellow images) lacked dendritic morphology. (d) Cells coexpressing IgE and CD11c are absent in normal skin from a non-atopic volunteer. Nuclei are labeled with DAPI (blue images). Original magnification: ×100.

Double immunofluorescence revealed that some infiltrating IgE+ cells coexpressed CD11c in the lichenified lesions of AD in Cases 1 and 2. The majority of IgE+CD11c+ cells showed a dendritic morphology and were distributed in the papillary and upper dermis, accompanying both IgE+CD11c-and IgE-CD11c+ cells (Figs. 3a, 4a). Only a few dendritic IgE+CD11c+ cells were seen in the lower epidermis of Case 2 (Fig. 3a). Although IgE+CD11c-and IgE-CD11c+ cells existed in small to moderate numbers, IgE+CD11c+ cells were absent in specimens from Case 4 or any control cases of non-atopic chronic eczema (Figs. 3b, 4b). IgE+CD11c+ cells were also present with regular distribution among dermis-infiltrating cells in cured normal-appearing skin from Case 3, but most had lost the dendritic morphology (Fig. 4c). Those cells coexpressing IgE and CD11c were absent from the normal skin of a non-atopic volunteer (Fig. 4d).

## Discussion

Genetic susceptibilities and the host environment play important roles in AD, and the pathogenesis is characterized by IgE-mediated allergy and skin barrier defects.^9^ However, the contribution of allergen-specific IgE to the eczematous lesion remains controversial.^10^ The present study used immunohistochemical and double immunofluorescence studies to demonstrate numerous IgE-bearing cells in the inflammatory infiltrate of lichenified lesions in the extrinsic (IgE-allergic) form of elderly AD (Cases 1–3). The majority of infiltrating IgE+ cells were either mast cells or CD11c+ dermal dendritic cells. Those IgE+ mast cells and IgE+CD11c+ dermal dendritic cells were observed in every onset type of extrinsic elderly AD. Although mast cell numbers and IgE production are affected by several factors, such as anatomical skin sites,^11^ corticosteroids used in treatment^12^,^13^ and chronic non-specific irritation,^14^ we think that the results in our study can be considered to reflect our experimental objectives.

Contrasting with previous reports^15^–^21^ that indicated the presence of high-affinity receptor for IgE (FcεRI) and cell-surface binding IgE on CD1a+ dermal dendritic cells, macrophages and eosinophils in lesional AD skin, only these few IgE+ cells were identified in the present study. IgE+ CD1a+ Langerhans cells in the epidermis were found as small numbers only in the frozen sections. We speculate that this discrepancy may be related to differences in the materials used and methods applied in the present practical analysis (e.g. use of specimens from skin lesions without a therapy-free interval, anti-IgE mAb for both paraffin-embedded and frozen sections, and basic evaluation by single immunohistochemical labeling with serial paraffin sections) compared to previous studies.^22^,^23^

Immunohistochemical and double immunofluorescence studies also revealed the presence of IgE+ cells in the cured normal-looking skin of a patient with extrinsic elderly AD after successful treatment (Case 3), although serum levels of total IgE and specific IgEs had decreased to within normal ranges. Interestingly, not only the IgE+ mast cells, but also the IgE+CD11c+ cells were regularly distributed in the dermis of normalized skin. These findings suggest that IgE-dependent, non-mast-cell-mediated immune pathways may also be prepared in the non-inflammatory skin of AD patients.^17^

In the one case with intrinsic-form (without detectable IgE-mediated sensitization) elderly AD (Case 4), we could not identify any positive findings indicating the involvement of IgE-bearing cells. A previous investigation proposed that part of intrinsic AD may be a transitional form of extrinsic AD with latent local IgE-allergy in the skin,^24^ because the allergic form of AD sometimes changes from intrinsic to extrinsic over time, particularly in young children.^25^ Other researchers have presumed that patients with intrinsic AD are not sensitized with typical protein environmental allergens, which induce Th2 responses and IgE-allergy, but rather with other antigens, e.g. metals and staphylococcal superantigens inducing Th1 or mixed Th1/Th2 responses.^26^ Given our results for Cases 3 and 4, we speculate that the intrinsic form of elderly AD may comprise different subtypes.^4^

In conclusion, we demonstrated that infiltrating IgE+ cells mostly comprise mast cells and CD11c+ dermal dendritic cells, both in lesional lichenified skin and in cured normal-appearing skin of patients with extrinsic elderly AD. These results suggest that, similar to other age-groups of AD, IgE-mediated allergic inflammation may play an important role in the pathobiology of elderly AD.

## References

[1] Tanei R, Katsuoka K (2008). Clinical analyses of atopic dermatitis in the aged. J Dermatol.

[2] Tanei R (2009). Atopic dermatitis in the elderly. Inflamm Allergy Drug Targets.

[3] Katsarou A, Armenaka MC (2011). Atopic dermatitis in older patients: particular points. J Eur Acad Dermatol Venereol.

[4] Bozek A, Fisher A, Filipowska B (2012). Clinical features and immunological markers of atopic dermatitis in elderly patients. Int Arch Allergy Immunol.

[5] Hanifin JM, Rajka G (1980). Diagnostic features of atopic dermatitis. Acta Dermatovener (Stockh).

[6] (1993). European Task Force on Atopic Dermatitis. Severity scoring of atopic dermatitis: the SCORAD index. Dermatology.

[7] Saeki H, Furue M, Furukawa F (2009). Guidelines for management of atopic dermatitis. J Dermatol.

[8] Sawabe M, Hamamatsu A, Ito T (2003). Early pathogenesis of cardiac amyloid deposition in senile systemic amyloidosis: close relationship between amyloid deposits and the basement membranes of myocardial cells. Virchows Arch.

[9] Werfel T (2009). The role of leukocytes, keratinocytes, and allergen-specific IgE in the development of atopic dermatitis. J Invest Dermatol.

[10] Flohr C, Johansson SGO, Wahlgren CF, Williams H (2004). How atopic is atopic dermatitis?. J Allergy Clin Immunol.

[11] Weber A, Knop J, Maurer M (2003). Pattern analysis of human cutaneous mast cell populations by total body surface mapping. Br J Dermatol.

[12] Pipkorn U, Hammarlund A, Enerbäck L (1989). Prolonged treatment with topical glucocorticoids results in an inhibition of the allergen-induced weal-and-flare response and a reduction in skin mast cell numbers and histamine content. Clin Exp Allergy.

[13] Jabara HH, Brodeur SR, Geha RS (2001). Glucocorticoids upregulate CD40 ligand expression and induced CD40L-dependent immunoglobulin isotype switching. J Clin Invest.

[14] Allakhverdi Z, Comeau MR, Jessup HK (2007). Thymic stromal lymphopoietin is released by human epithelial cells in response to microbes, trauma, or inflammation and potently activates mast cells. J Exp Med.

[15] Ochoa MT, Loncaric A, Krutzik SR (2008). ‘Dermal dendritic cells’ comprise two distinct populations: CD1 +  dendritic cells and CD209 +  macrophages. J Invest Dermatol.

[16] Guttman-Yassky E, Lowes MA, Fuentes-Duculan J (2007). Major differences in inflammatory dendritic cells and their products distinguish atopic dermatitis from psoriasis. J Allergy Clin Immunol.

[17] Leung DYM, Schneeberger EE, Siraganian RP (1987). The presence of IgE on macrophages and dendritic cells infiltrating into the skin lesion of atopic dermatitis. Clin Immunol Immunopathol.

[18] Tanaka Y, Takenaka M, Matsunaga Y (1995). High affinity IgE receptor (FcεRI) expression on eosinophils infiltrating the lesions and mite patch tested sites in atopic dermatitis. Arch Dermatol Res.

[19] Klubal R, Osterhoff B, Wang B (1997). The high-affinity receptor for IgE is the predominant IgE-binding structure in lesional skin of atopic dermatitis patients. J Invest Dermatol.

[20] Bruynzeel-Koomen C, Wichen DF, Toonstra J (1986). The presence of IgE molecules on epidermal Langerhans cells in patients with atopic dermatitis. Arch Dermatol Res.

[21] Bieber T, Dannenberg B, Prinz JC (1989). Occurrence of IgE-bearing epidermal Langerhans cells in atopic eczema: a study of the time course of the lesions and with regard to the IgE serum level. J Invest Dermatol.

[22] Bieber T, Ring J, Rieber EP (1988). Anti-IgE monoclonal antibodies as tools for demonstration of cutaneous IgE bearing dendritic cells. J Invest Dermatol.

[23] Stary G, Bangert C, Stingl G, Kopp T (2005). Dendritic cells in atopic dermatitis: expression of FcεRI on two distinct inflammation-associated subsets. Int Arch Allergy Immunol.

[24] Novak N, Bieber T (2003). Allergic and nonallergic forms of atopic diseases. J Allergy Clin Immunol.

[25] Park JH, Choi YL, Namkung JH (2006). Characteristic of extrinsic vs. intrinsic atopic dermatitis in infancy: correlations with laboratory variables. Br J Dermatol.

[26] Tokura Y (2010). Extrinsic and intrinsic types of atopic dermatitis. J Dermatol Sci.

